# Analysis of Vascular Permeability by a Modified Miles Assay

**DOI:** 10.21769/BioProtoc.5264

**Published:** 2025-04-05

**Authors:** Hilda Vargas-Robles, Karina B. Hernández-Almaraz, Michael Schnoor

**Affiliations:** Department for Molecular Biomedicine, Centro de Investigación y de Estudios Avanzados del Instituto Politécnico Nacional (Cinvestav-IPN), Mexico-City, Mexico

**Keywords:** Endothelium, Vascular permeability, Evans blue, Histamine, Dimethylformamide

## Abstract

The endothelial barrier is a semipermeable cell layer covering the inside of blood vessels that regulates the flux of ions, macromolecules, and plasma from blood to tissues. Inflammation promotes an increase in vascular permeability, which can contribute to disease if not controlled properly. Thus, it is important to understand in detail the molecular mechanisms underlying inflammatory vascular hyperpermeability. While endothelial permeability can be measured in vitro, these assays do not recapitulate precisely the in vivo vasculature. Thus, in vivo assays are required to understand the full picture of vascular permeability regulation. Here, we describe an established assay that involves injection of Evans blue dye followed by intradermal injection of agents inducing vascular permeability. This assay is relatively easy to perform and provides reliable data on permeability regulation in vivo.

Key features

• Step-by-step protocol to study vascular permeability in the mouse skin.

• Injection of Evans blue dye followed by intradermal injection of permeability-inducing agents allows reproducible analysis of regulatory mechanisms.

• This protocol allows the analysis of different substances in the same animal.

• Possibility of different dye administration routes that can be compared.

## Graphical overview


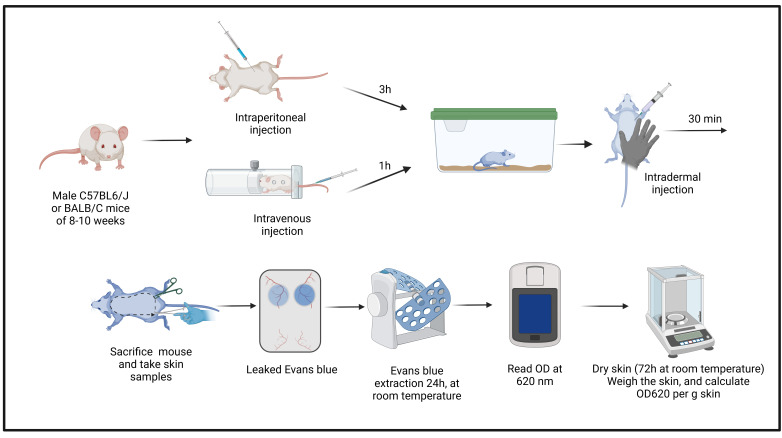


## Background

The endothelium is a monolayer of endothelial cells (ECs) that constitutes the inner cellular lining of all blood vessels including arteries, veins, and capillaries. Endothelial cells form a semipermeable barrier that regulates the passage of plasma proteins and circulating cells from blood to tissues. This function occurs via paracellular and transcellular flux, with the paracellular route being the predominant one. Paracellular flux occurs across interendothelial cell contacts that are sealed by adhesive interactions between transmembrane protein complexes. These adhesive interactions are provided by tight junctions (TJs)—formed by proteins such as occludin, claudins, JAMs (junction-associated molecules), and zonula occludens adaptor proteins—and adherent junctions (AJs), formed by VE-cadherin and scaffold proteins of the catenin family. TJs and AJs are bound to the actin cytoskeleton to provide physical strength [1]. The actin cytoskeleton plays an important role in regulating the stability of endothelial cell junctions and thus vascular permeability [2]. The actin cytoskeleton is a dynamic structure that is remodeled in response to different stimuli via proteins of the Rho GTPases family and actin-binding proteins such as the Arp2/3 complex, cortactin, formins, and cofilin [3,4]. Actin remodeling is key to vascular permeability regulation, and different types of actin filaments can either stabilize the EC barrier and reduce permeability or destabilize the barrier to increase permeability (hyperpermeability), e.g., in the case of actin stress fiber formation [5,6].

Vascular hyperpermeability is induced during inflammation, which is important to allow blood components to reach the inflamed site and contribute to resolution and healing. Factors increasing permeability include histamine, bradykinin, thrombin, and platelet-activating factor (PAF), among many others. For example, histamine and bradykinin promote Y685 phosphorylation in VE-cadherin to increase vascular permeability [7,8], whereas thrombin promotes junction opening and permeability through Rho GTPase activation [9], leading to the formation of actomyosin contractility, junction disassembly, and consequent hyperpermeability [10].

Given the complexity of EC barrier regulation and its importance for inflammation and inflammatory diseases, it is critical to better understand the molecular mechanisms regulating vascular permeability to discover novel approaches to manipulate the EC barrier and thus ameliorate disease symptoms associated with vascular hyperpermeability. Although different in vitro methods exist (e.g., measurement of transendothelial electrical resistance (TEER) and paracellular macromolecular flux across EC cultivated on trans-well filters), they do not mimic entirely the in vivo situation since blood vessels are formed by many components other than EC and are in contact with other cells and the extracellular matrix. TEER measurements are also prone to variations due to microbubbles in the wells, electrode positioning, and changes in the pH of the medium.

The relevance of the study of EC barrier regulation for health and disease in vivo was recognized decades ago. In 1952, Miles and Miles developed a simple assay based on dye administration into the bloodstream to study its leakage in vivo under different conditions [11]. Over time, different dyes have been evaluated for the analysis of endothelial permeability [12]. Evans Blue has emerged as the dye of choice as it can be easily extracted and quantified, which is a major advantage over the original Miles assay that used Pontamine blue and relied on changes in size and intensity of the stained skin for evaluation [11]. Evans blue dye has a high affinity for serum albumin, circulates easily throughout the body without toxic effects, and can be easily quantified once leaked from the blood into tissue [13]. Here, we present in detail this modified protocol that permits the application of different compounds in the same animal to study their local effects on vascular permeability simultaneously.

## Materials and reagents

**Biological materials**

1. Male, 8–10-week-old C57BL6/J and BALBC mice (animal facility of Cinvestav-IPN)

**Reagents**

1. Evans blue 2%, intraperitoneal (IP); 0.5%, intravenous (IV) (Merck, catalog number: E2129)

2. Histamine dihydrochloride (Merck, catalog number: 53300)

3. Saline solution 0.9% (PiSA, catalog number: 82175)

4. Dimethylformamide (J.T. Baker, catalog number: 9221-02)

**Caution:** This chemical is corrosive.

5. Ketamine hydrochloride 100 mg/mL (Aranda, catalog number: Q-0449-265)

6. Xylazine 20 mg/mL (PROCIN^®^, PiSA, Reg Q-7833099)

7. Ethyl alcohol (J.T. Baker, catalog number: 9000-03)

8. Sodium chloride (NaCl) (J.T. Baker, catalog number: 3624-05)

9. Potassium chloride (KCl) (J.T. Baker, catalog number: 3040-01)

10. Sodium phosphate dibasic anhydrous (Na_2_HPO_4_) (J.T. Baker, catalog number: 3828-01)

11. Potassium dihydrogen phosphate (KH_2_PO_4_) (J.T. Baker, catalog number: 3252-01)

12. Milli-Q water (Millipore, obtained using a Milli-Q reference device)

**Solutions**

1. Phosphate buffered solution (PBS) 1× (see Recipes)

2. Anesthesia (see Recipes)

3. Evans blue 2% and 0.5% (see Recipes)

4. Histamine solution (see Recipes)

5. Alcohol 70% (see Recipes)

**Recipes**

**1. Phosphate buffered solution (PBS) 1×**

**Table:** 

Reagent	Final concentration	Quantity or Volume
NaCl	138 mM	8.06 g
KCl	3 mM	0.22 g
Na_2_HPO_4_	8.1 mM	1.15 g
KH_2_PO_4_	1.5 mM	0.20 g
Milli-Q H_2_O	n/a	1,000 mL
Total	n/a	1,000 mL

**2. Anesthesia solution [IP dosing amount (12.5 μL/g mouse weight)]**

**Table:** 

Reagent	Final concentration	Quantity or Volume
Ketamine hydrochloride	100 mg/kg	150 μL
Xylazine	13 mg/kg	75 μL
Saline solution	n/a	1,275 μL
Total	n/a	1,500 μL

**3. Evans blue 2% and 0.5%**

**Table:** 

Reagent	Final concentration	Quantity or Volume
Evans blue	2% (w/v) 0.5% (w/v)	2 g 0.5 g
1× PBS	n/a	100 mL
Total	n/a	100 mL

**4. Histamine solution**

**Table:** 

Reagent	Final concentration	Quantity or Volume
Histamine	20 ng/μL (w/v)	1,000 ng
PBS 1×	n/a	50 μL
Total	n/a	50 μL

**5. Alcohol 70%**

**Table:** 

Reagent	Final concentration	Quantity or Volume
Ethyl alcohol	70% (v/v)	70 mL
Milli-Q H_2_O	n/a	30 mL
Total	n/a	100 mL

**Laboratory supplies**

1. Insulin syringes, 27G × 13 mm gauge (G) needles, 1 mL (BD Plastipak, catalog number: 326716)

2. Syringe filter 0.22 μm (TPP, catalog number: 99722)

3. Microtubes (Axygen, catalog number: MCT-150-C)

4. 1,000 μL pipette tips (Axygen, catalog number: T-1000-B)

5. 200 μL pipette tips (Axygen, catalog number: T-200-Y)

6. 10 μL pipette tips (Axygen, catalog number: T-300)

7. Microtubes 2 mL (Costar Corning, catalog number: 10276141)

8. Eppendorf UVett (Eppendorf, catalog number: 0030106318)

## Equipment

1. Safe Aire II chemical hood (Thermo Scientific, catalog number: FH3943810244)

2. Biological safety cabinet, class II, type A2 (Nuaire, catalog number: NU-540-400)

3. Tweezers kit, tip stile 2A (Agar Scientific, catalog number: AGT5530)

4. Dissecting scissors (10.5 cm length) (Fine Science Tools, 14160-10)

5. Kelly hemostats (14 cm length) (Fine Science Tools, 13018-14)

6. Rotator AG (Daigger, Seoul, South Korea, model: AG)

7. Biophotometer (Eppendorf, model: 6131)

8. Adventurer Analytical Balance, OHAUS V11140 (Ohaus Corporation, model: 30100601)

9. 4-way tube racks (Heathrow Scientific, catalog number: HS29022G)

10. Electric shaver (Oster, model: 78005-010)

## Software and datasets

1. GraphPad, version 8.0.1 (requires license)

2. BioRender (https://www.biorender.com/). The following figures were created using BioRender: Graphical overview, https://BioRender.com/s23o513; Figure 6, https://BioRender.com/c90a101

## Procedure

**A. Evans blue intraperitoneal (IP) administration (2% Evans blue dye)**

1. Prepare the 2% Evans blue dye solution (see Recipes).

2. Homogenize the solution well by vortexing.

3. Transfer the solution into a 20 mL syringe and filter it through a 0.22 μm syringe filter. Keep the solution at room temperature.

4. Weigh the mice.

5. Use an insulin syringe with a 27G × 13 mm needle and fill with 2% Evans Blue dye at 4 mL/kg mouse body weight.

**Critical:** Carefully remove all bubbles in the syringe.

**Figure 1.**
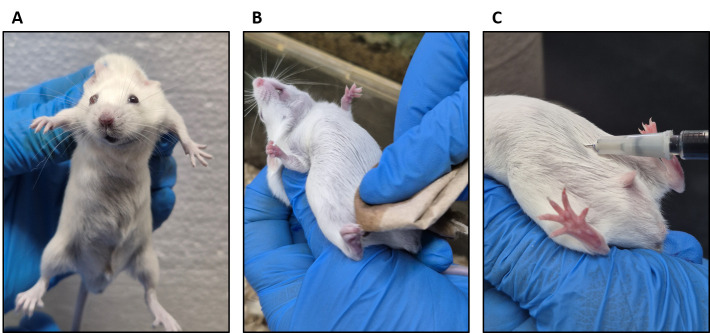
Evans blue intraperitoneal administration. (A) Holding the mouse. (B) Cleaning the abdominal area with ethyl alcohol. (C) Evans blue inoculation.

6. Hold the mouse gently by its tail. Then, with your free hand, gently grab its neck and turn the mouse to expose the abdomen (Figure 1A).

7. Clean the abdomen with tissue soaked in 70% ethanol where the injection will be applied (Figure 1B).

*Note: See the following video for administration guidelines:* *https://www.youtube.com/watch?v=s9skgg7dHIA&t=712s*

8. Insert the needle at a 45° angle. (Optional: Aspirate a little to ascertain that you are in the peritoneum.)

9. Slowly administer the Evans blue dye (Figure 1C).

10. Return the animal to its cage and let the dye absorb for 3 h.

**Figure 2.**
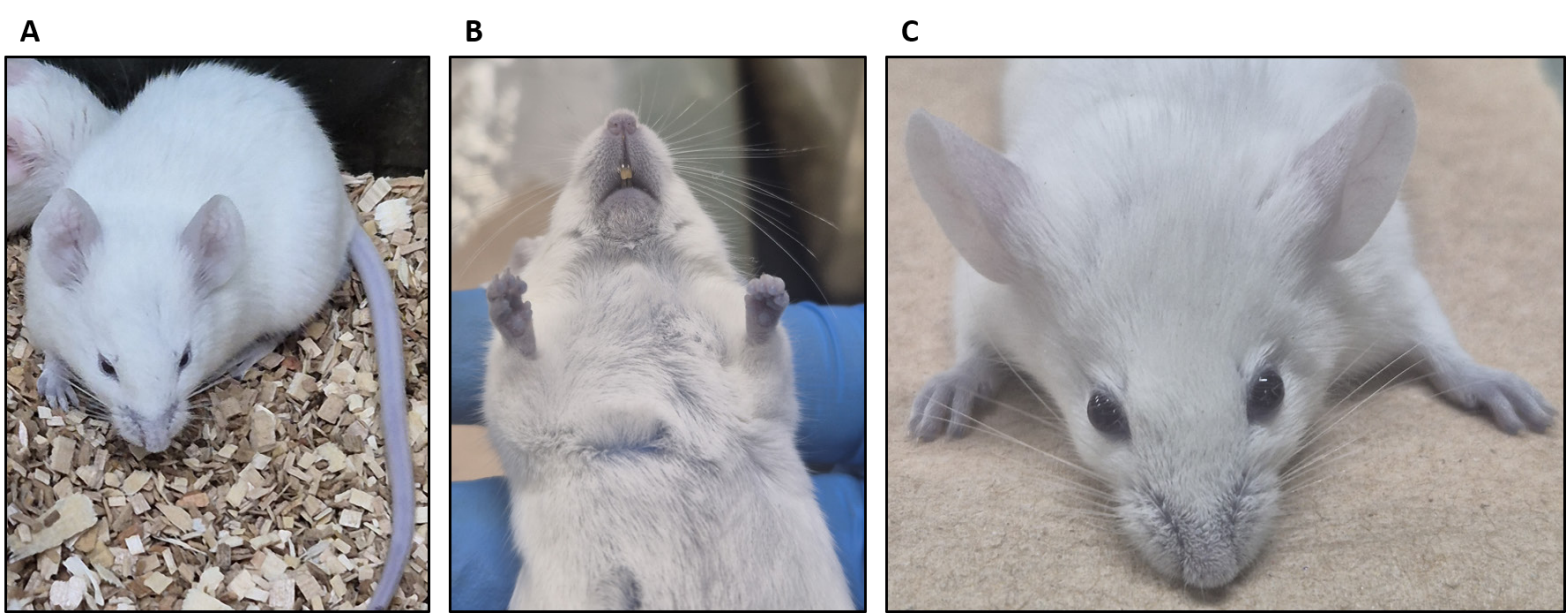
The BALB/C mouse appears slightly blue after 3 h of Evans blue dye administration. (A) General view of the mouse with slightly blue coloration. (B) Mouth and foot pads appear blue after Evans blue injection. (C) Ears appear slightly blue after Evans blue injection.

*Note: After 3 h, the footpads, nose, and ears must appear slightly blue indicating successful injection (Figure 2). The mouse is now ready for the permeability assay.*

**B. Evans blue intravenous (IV) administration (0.5% Evans blue dye)**

1. Prepare the 0.5% Evans Blue dye solution (see Recipes).

2. Homogenize the solution well by vortex.

3. Transfer the solution into a 20 mL syringe and filter it through a 0.22 μm syringe filter. Keep the solution at room temperature.

4. Weigh the mice.

5. Use a 27G × 13 mm syringe and fill the syringe with 100 μL of 0.5% Evans blue dye per mouse. Carefully remove all bubbles in the syringe.

6. Keep the mouse briefly (~5–10 min) under an infrared lamp to dilate the tail veins.

*Note: If no infrared lamp is available, you can incubate the tail briefly in 37 °C water.*

**Figure 3.**
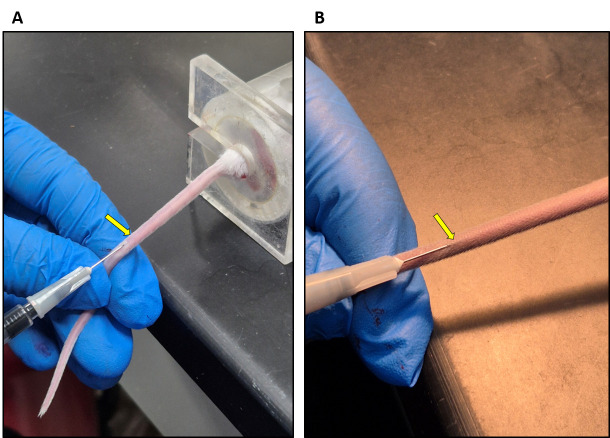
Evans blue injection into the tail vein. (A) BALB/C mouse. (B) C57BL6/J mouse. Arrows show the lateral vein.

7. Transfer the mouse gently into a mouse restrainer to immobilize it.

8. Clean the tail with 70% ethanol.

9. Insert the needle carefully into the lateral vein and slowly administer the Evans blue dye solution (Figure 3).

*Note: Intravenous injections are not easy and require practice. Make sure to hold the needle at a flat angle (see Figure 3) to enter the vein. If you sense any resistance while administering, you have not entered the vein correctly. The veins will be much easier to see in a BALB/C mouse (Figure 3).*

11. Return the animal to its cage and let the dye circulate for 30–60 min.

**Figure 4.**
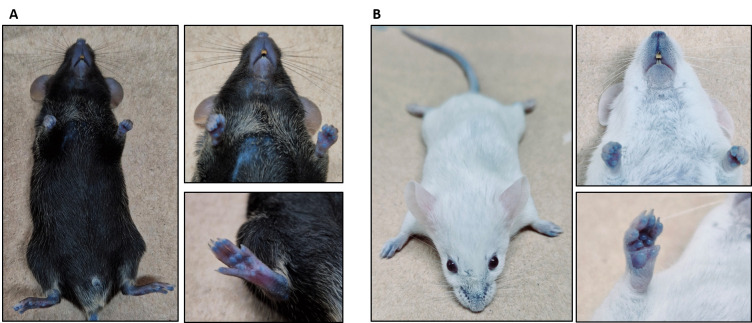
Evans blue body distribution. (A) C57BL6/J mouse. (B) BALB/C mouse. Please note that an intravenous (IV) injection leads to a more intense blue coloration than the intraperitoneal (IP) injection (compare with Figure 2).

*Note: Usually, after 30 min, the footpads, nose, and ears of the mouse will turn blue if the dye was injected properly. The mouse is now ready for the permeability assay (Figure 4).*

**C. Permeability assay**

1. Prepare the anesthesia solution (see Recipes).

2. Administer the anesthesia solution intraperitoneally as described above.

*Note: Wait for 10 min and then monitor the pain reflexes of the mouse by pinching the paws with forceps.*

**Figure 5.**
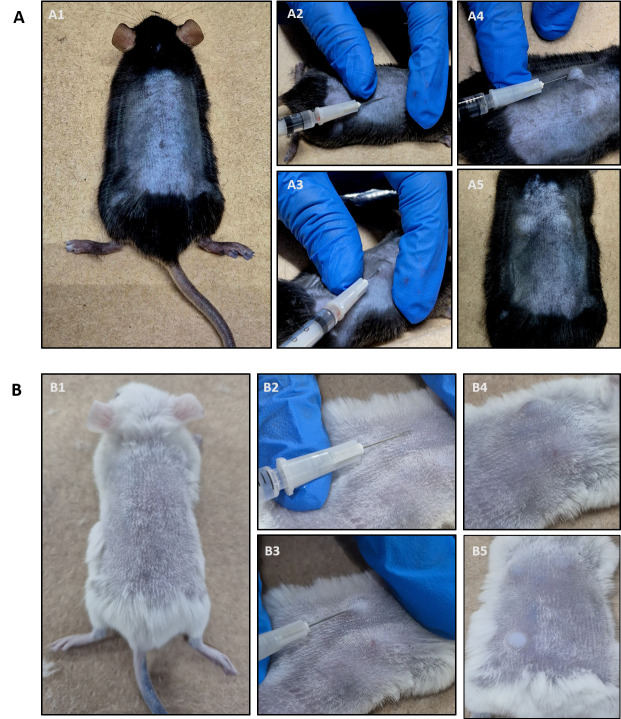
Intradermal histamine injection into the shaved back skin. (A) C57BL6/J mouse. (B) BALB/C mouse. After correct intradermal injection, skin bubbles are visible (A4/A5 and B4/B5). For a detailed description of each procedure please see the text.

3. Turn the mouse and shave the back carefully with a veterinary clipper shaver (Figure 5A1, B1).

*Note: It is advised to wait 1 h after shaving or shaving the day before the experiment since shaving irritates the skin.*

4. Prepare the sterile histamine solution (see Recipes).

5. Fill an insulin syringe with a 27G × 13 mm needle with the 1,000 ng/50 μL histamine solution.

6. Stretch the skin with your index finger and thumb (Figure 5A2/B2 and A3/B3).

7. Insert ~5 mm of the needle at a 45° angle carefully into the skin. Administer the histamine solution very slowly and continuously.

*Notes:*

*1. The administration must be intradermal. Successful intradermal injection is indicated by the formation of a skin bubble (Figure 5A4, A5 and B4, B5). If a bubble does not appear, you have likely injected subcutaneously and this injection site must be aborted. Intradermal injection is characterized by strong resistance when inserting the needle. If the needle moves without resistance, it is most likely a subcutaneous injection.*

*2. Repeat injections in the same mouse if you want to test different stimuli by always applying 50 μL of each substance approximately 1–2 cm away from another injection site (up to 6 injections are possible per mouse back).*

**Figure 6.**
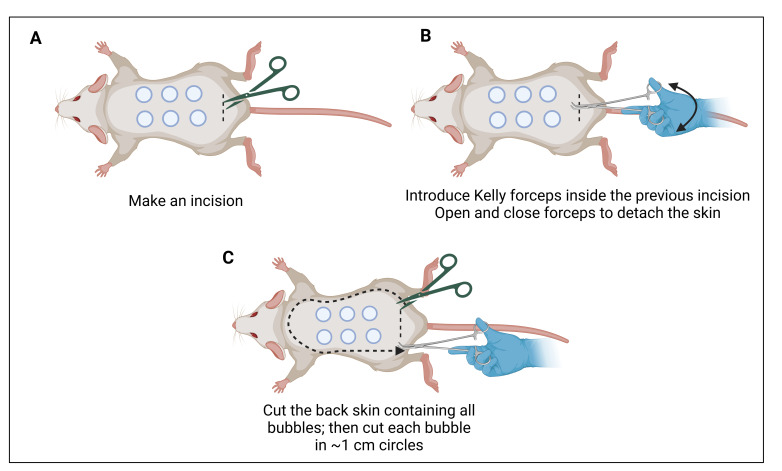
Skin cutting. (A) Make a transversal incision in the lower skin. (B) Kelly forceps are introduced between the skin layer and the muscular layer. (C) Once the skin is separated, it can be lifted and cut. Make sure to have good margins (~0.5 cm) along the injection sites.

**Figure 7.**
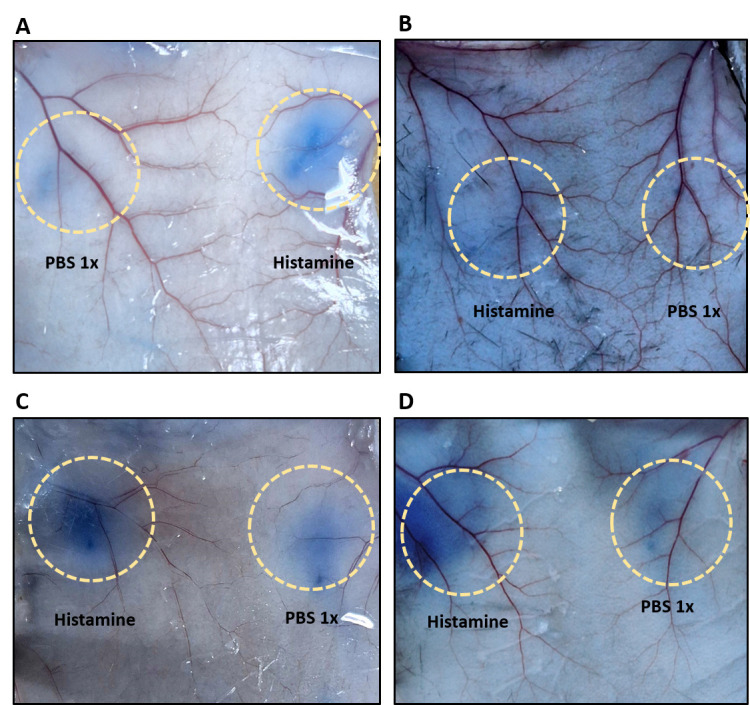
Evans blue leakage at the injection sites indicating vascular permeability. (A) After Evans blue intraperitoneal (IP) administration in a BALB/C mouse. (B) After Evans blue IP administration in a C57BL/6J mouse. (C) After Evans blue intravenous (IV) administration in a BALB/C mouse. (D) After Evans blue IV administration in a C57BL/6J mouse. Please note that leakage is more intense after IV injection, thus making this the preferred route of administration. Yellow circles highlight the site of injection.

8. Return the mouse to its cage and wait 30 min.

9. After 30 min, euthanize the anesthetized mouse by cervical dislocation or anesthesia overdose (triple dose of that mentioned above).

10. Make an incision in the lower skin using surgical scissors (Figure 6A).

11. Separate the skin from the muscle and tissue using Kelly forceps (Figure 6B).

12. Cut the entire back skin ensuring sufficient margins around the injection sites (Figure 6C).

13. Then, cut ~1 cm circles to recover the injected skin areas for dye extraction and quantification (Figure 7). *Note: The circles should be centered around the blue leakage as the injection site is not always clearly visible.*

14. Place skin samples in 1 mL of dimethylformamide in a 2 mL microtube.

15. Put the tube in an overhead rotator at 40 rpm at room temperature for 24 h.

16. Place 100 μL of dimethylformamide as blank and 100 μL of each sample supernatant (extracted dye) in a cuvette or microplate and read the absorption spectrophotometrically at 620 nm.

17. Take out the skin samples from the Eppendorf tube and dry the skin circles at room temperature for 2 days at room temperature on the workbench

18. Weigh every sample on an analytical scale.

## Data analysis

**Figure 8.**
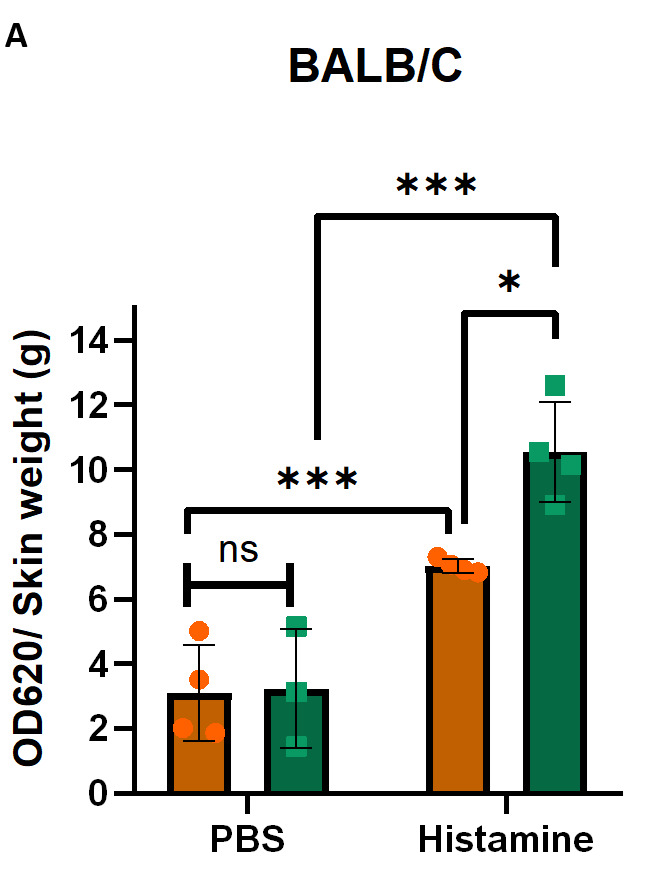
Evans blue leakage in the skin of BALB/c and C57BL6/J mice. (A) Quantification of Evans blue leakage after intraperitoneal (IP) and intravenous (IV) administration with PBS as control or histamine in BALB/c mice. (B) Quantification of Evans blue leakage after IP and IV administration with PBS or histamine in C57BL6/J mice. Error bars represent the standard deviation of the mean. As we compared two different variables in two different groups, two-way ANOVA was used to determine statistical significance; n = 2–4 per group; individual data points are shown in the bars; *p **≤** 0.05; ***p **≤** 0.001; ns, non-significant.

After measuring the optical density (OD) spectrophotometrically at 620 nm and determining the dry weight of each skin sample, calculate the OD per gram of tissue (OD_620_/g dry skin) and prepare a graph using GraphPad software as shown in Figure 8, which displays representative data in BALB/C (Figure 8A) and C57BL6/J (Figure 8B) mice. Data for both IP and IV administration of Evans blue are included. Please note that the OD_620_ after IV administration is much higher than after IP administration. IV is therefore the preferred route. Different substances can be tested for their ability to regulate vascular permeability. Depending on the type of substance and expected effect, they can be applied either together with the Evans blue IP or IV, or alone or in combination with other substances into the skin. It is important to always include PBS and histamine-only controls to be sure that the assay worked properly and that you are observing the true effects of your substances of interest.

## Validation of protocol

This protocol or parts of it has been used and validated in the following research articles:

• Montoya-García et al. [5]. Arpin deficiency increases actomyosin contractility and vascular permeability. *eLife* (Figure 8E).

• Moztarzadeh et al. [6]. Erk1/2 is not required for endothelial barrier establishment despite its requirement for cAMP-dependent Rac1 activation in heart endothelium. Arpin deficiency increases actomyosin contractility and vascular permeability. *Tissue Barriers* (Figure 6A and B).

## General notes and troubleshooting

**General notes**

1. Avoid air conditioning when performing the assay as temperature changes affect skin permeability as long as the mouse is alive.

2. Reagents must be prepared in a class II biosafety cabinet and solutions should be filtered using a 0.22 μm filter.

3. Maintain sterile conditions for stock solutions.

4. Evans blue binds to albumin and globulins in the blood. Thus, differently bound Evans blue forms may leak differently. If such size variations are of relevance for certain experiments, leakage of differently sized FITC-dextrans can be tested.

**Troubleshooting**

Problem 1: Variability in the same mouse and between different mice of the same group.

Possible cause: Application of the intradermal injection in different skin areas where branched veins are very different.

Solution: Try to inject in the same areas from the middle of the back and compare OD values according to the same skin area.

Problem 2: Variability between IP and IV Evans blue administration.

Possible cause: IP-injected Evans blue needs to be absorbed, while Evans blue via IV directly enters circulation and immediately binds to serum albumin. Thus, different circulation times and amounts can affect the result.

Solution: If possible, use BALB/C mice, in which IV administration is easy and reproducible because of better visibility of the lateral tail veins compared to C57BL6/J mice.

Problem 3: Variability of IP Evans blue administration.

Possible cause: Fat affects absorption.

Solution: Use mice of the same weight and age.
